# The role of albumin and the extracellular matrix on the pathophysiology of oedema formation in severe malnutrition

**DOI:** 10.1016/j.ebiom.2022.103991

**Published:** 2022-04-07

**Authors:** Gerard Bryan Gonzales, James M. Njunge, Bonface M Gichuki, Bijun Wen, Moses Ngari, Isabel Potani, Johnstone Thitiri, Debby Laukens, Wieger Voskuijl, Robert Bandsma, Jill Vanmassenhove, James A Berkley

**Affiliations:** a Nutrition, Metabolism and Genomics Group, Division of Human Nutrition and Health, Wageningen University and Research, Wageningen, the Netherland; b Department of Internal Medicine and Paediatrics, Laboratory of Gastroenterology, Faculty of Medicine and Health Sciences, Ghent University, Ghent, Belgium; c VIB-UGent Center for Inflammation Research, Ghent, Belgium; d The Childhood Acute Illness & Nutrition (CHAIN) Network, Nairobi, Kenya; e KEMRI/Wellcome Trust Research Programme, Kilifi, Kenya; f Centre for Global Child Health, The Hospital for Sick Children, Toronto, Ontario, Canada; g Department of Nutritional Sciences, Faculty of Medicine, University of Toronto, Toronto, Canada; h Kamuzu University of Health Sciences (Former College of Medicine), Blantyre, Malawi; i Amsterdam Centre for Global Child Health, Emma Children's Hospital, Amsterdam University Medical Centres, Amsterdam, the Netherland; j Department of Global Health, Amsterdam Institute for Global Health and Development, Amsterdam University Medical Centres, Amsterdam, the Netherland; k Department of Internal Medicine and Paediatrics, Renal Division, Faculty of Medicine and Health Sciences, Ghent University, Ghent, Belgium; l Nuffield Department of Medicine, Centre for Tropical Medicine & Global Health, University of Oxford, Oxford, UK

**Keywords:** Starling forces, Extracellular matrix, Endothelial glycocalyx, Oedema, Proteomics, Severe malnutrition

## Abstract

**Background:**

While fluid flows in a steady state from plasma, through interstitium, and into the lymph compartment, altered fluid distribution and oedema can result from abnormal Starling's forces, increased endothelial permeability or impaired lymphatic drainage. The mechanism of oedema formation, especially the primary role of hypoalbuminaemia, remains controversial. Here, we explored the roles of albumin and albumin-independent mechanisms in oedema formation among children with severe malnutrition (SM).

**Methods:**

We performed secondary analysis of data obtained from two independent clinical trials in Malawi and Kenya (NCT02246296 and NCT00934492). We then used an unconventional strategy of comparing children with kwashiorkor and marasmus by matching (discovery cohort, n = 144) and normalising (validation cohort, n = 98, 2 time points) for serum albumin. Untargeted proteomics was used in the discovery cohort to determine plausible albumin-independent mechanisms associated with oedema, which was validated using enzyme-linked immunosorbent assay and multiplex assays in the validation cohort.

**Findings:**

We demonstrated that low serum albumin is necessary but not sufficient to develop oedema in SM. We further found that markers of extracellular matrix (ECM) degradation rather than markers of EG degradation distinguished oedematous and non-oedematous children with SM.

**Interpretation:**

Our results show that oedema formation has both albumin-dependent and independent mechanisms. ECM integrity appears to have a greater role in oedema formation than EG shedding in SM.

**Funding:**

Research Foundation Flanders (FWO), Thrasher Foundation (15122 and 9403), VLIR-UOS-Ghent University Global Minds Fund, Bill & Melinda Gates Foundation (OPP1131320), MRC/DfID/Wellcome Trust Global Health Trials Scheme (MR/M007367/1), Canadian Institutes of Health Research (156307), Wellcome Trust (WT083579MA).

**Research in context**

**Evidence before this study**

Oedema formation, especially in severe malnutrition, is commonly attributed to hypoalbuminaemia associated with increased inflammation and vascular permeability, as explained by the Starling model. However, a revision of this principle was proposed after mounting evidence that the classical Starling model does not accurately predict fluid behaviour. The revised Starling model places emphasis on the contributions of the endothelial glycocalyx, the endothelial basement membrane, and the extracellular matrix and fluid balance depends on lymphatic function. It is understood that oedema develops when there are alterations in fluid movement from the intravascular, through the interstitium, and into the lymphatic compartments. The role of the intravascular, interstitium, and lymph compartments in oedema formation in kwashiorkor (hypoalbuminemia) has not been characterised.

**Added value of this study**

In the current study, we demonstrate that oedema in severe malnutrition is associated with both albumin-dependent and independent mechanisms. That is, albumin is necessary but not sufficient to cause oedema. Moreover, extracellular matrix degradation, but not endothelial glycocalyx degradation, is associated with oedema in severe malnutrition. We hypothesise that endothelial glycocalyx degradation is associated to disease severity but not oedema formation in oedematous diseases. We also provide novel data implicating the lymphatic system, in interstitial fluid accumulation in severe malnutrition.

**Implications of all the available evidence**

The mechanism of oedema formation, especially the primary role of hypoalbuminaemia, remains controversial and has major implications in the management of oedematous diseases including kwashiorkor. Our results highlight that managing hypoalbuminemia in kwashiorkor may not solve oedema because other factors in addition to hypoalbuminemia play vital role in its pathophysiology. We also highlight the need for deeper understanding of the role of the interstitium and lymph compartments in body fluid distribution.

Alt-text: Unlabelled box

## Introduction

The mechanisms of oedema formation is a subject of prolonged debate,^1^, ^2^, ^3^ including in childhood severe malnutrition (SM). The widely understood mechanism is that a reduction in plasma albumin, and hence colloid osmotic pressure (COP), reduces the intravascular-to-interstitial albumin gradient increasing fluid filtration from the intravascular compartment into the interstitial compartment, as explained by Starling forces^4^^,^^5^ as first described in 1896.^6^ However, in 2010 a revision of this principle was proposed by Levick and Michel^7^ after mounting evidence that the classical Starling model does not accurately predict fluid behaviour.^7^^,^^8^ For instance, intravenous fluid therapy does not result in extracellular volume expansion as expected in the classical model.^8^ Moreover, in hypoalbuminaemic states such as nephrotic syndrome, evidence suggests that factors beyond a decline in intravascular COP are responsible for altered fluid distribution.^9^

The revised Starling model highlights the contributions of the endothelial glycocalyx (EG) layer, endothelial basement membrane and the extracellular matrix (ECM) in fluid distribution. Most importantly, it posits that transcapillary flow (*J*_v_) is determined by the gradient between the intravascular COP (*π*_c_) and the COP within the sub-glycocalyx space (*π*_sg_), which is mostly protein-free, instead of the interstitial COP (*π*_is_). As the sub-glycocalyx space is low in protein, the COP across the glycocalyx opposes, but does not reverse *J*_v_ and hence the filtered fluid returns back to circulation mostly via the lymphatic system.^8^ Mortimer and Levick emphasize that oedema develops when the filtration rate which is governed by the Starling principle of fluid exchange exceeds lymph drainage for sufficient periods.^10^

We aimed to understand the mechanisms driving oedema formation among children suffering from severe malnutrition (SM) requiring hospitalisation (complicated SM). The syndrome of kwashiorkor (oedematous SM) is a striking phenotype of childhood SM comprising oedema, fatty liver, hair depigmentation, a desquamating skin rash and behavioural changes (Briend, www.ennonline.net/kwashiorkorstillanenigma). It is distinct from the syndrome of marasmus (severe wasting), characterised by low weight-for-height (<-3 Z-scores, 2006 WHO standards), low (<115mm) mid-upper arm circumference (MUAC), visible atrophy and loose skin.

Following the classical Starling model, the earliest and most widely accepted explanation of kwashiorkor was a low protein diet leading to hypoalbuminaemia, causing pathognomonic oedema.^11^ However, reports on the association between protein intake and kwashiorkor are conflicting.^12^, ^13^, ^14^, ^15^ Furthermore, a study observed that oedema in SM resolved independently of protein intake^2^ and without substantial increase in serum albumin.^3^ The latter claim was however questioned after reanalysis of the data^3^ suggesting that serum albumin had increased during oedema resolution.^1^ An examination of 10 studies showed increased plasma albumin following feeding in children with kwashiorkor.^1^ A further meta-analysis has also shown that circulating albumin is lower in kwashiorkor compared to marasmus.^16^ However, as we later show in this study, many children with SM present with low serum albumin concentrations, yet only some of these children develop oedema.

The role of albumin in the aetiology and pathophysiology of oedema in SM has been a subject of debate: one side ascribes hypoalbuminaemia as the primary mechanism for kwashiorkor^1^ while another side rejects it.^17^ It was previously reported that intestinal biopsies of children with kwashiorkor had lower expression and levels of sulphated glycosaminoglycans (GAGs), a main component of the glycocalyx, compared to those from children with marasmus,^18^ consistent with the revised Starling model. However, congenital conditions linked with the inability to produce GAGs are not typically associated with oedema formation^19^^,^^20^ (www.ennonline.net/kwashiorkorstillanenigma).

Comparing oedematous and non-oedematous children with SM with similar serum albumin levels presents an opportunity to understand mechanisms involved in oedema formation that operate independently of albumin and may shed light on mechanisms driving fluid distribution in support of or beyond Starling forces. We investigated albumin-independent mechanisms in the pathophysiology of oedema using children with SM as a proof-of-principle.

## Methods

### Overall study design

This study comprised of two separate nested case-control analyses of subsets of children with either kwashiorkor or marasmus from two clinical trials in Malawi and Kenya, as further described below. A hypothesis generating discovery cohort was used to explore albumin-independent mechanisms, which were then validated using the second cohort.

### Study population and setting

#### Discovery cohort

The discovery cohort was nested within a randomised controlled trial (NCT02246296) that aimed at determining the effect of a lactose-free, low-carbohydrate F-75 milk formulated to limit carbohydrate malabsorption, diarrhoea and refeeding syndrome among children hospitalized for complicated SM in Queen Elizabeth Central Hospital in Malawi, and Kilifi County Hospital and Coast General Teaching and Referral Hospital in Kenya.^21^ The trial enrolled children aged 6 months to 13 years at admission to hospital with complicated SM, defined as: mid-upper arm circumference (MUAC) <11.5 cm or weight-for-height Z score <−3 if younger than 5 years of age, body-mass-index Z score <−3 if older than 5 years, or oedematous malnutrition at any age. The children were predominantly admitted to hospital because of medical complications, and a few (8/843, 0.9%) for a failed appetite test as defined by WHO guidelines (http://apps.who.int/iris/bitstream/10665/41999/1/a57361.pdf). Children were excluded if they had a known allergy to milk products or consent was withheld. The primary outcome of the trial was the time to initial stabilization, defined as having reached the ‘transition’ phase of SM care and switched to a standard higher-caloric feed based on WHO guidelines. Biological samples for research including serum and plasma samples were collected upon admission but before randomization and stored at -80°C until analysis. For the trial, biochemical tests were performed, including determining serum albumin concentration. Clinical findings were also recorded such as presence of shock, pneumonia, malaria, heart disease, cerebral palsy and diarrhoea, as well as breastfeeding. The trial recruited a total of 843 children of which 8.9% died prior to stabilization and another 6.2% died after the first stabilization.^21^

#### Validation cohort

The validation cohort was nested within a randomised controlled trial (NCT00934492) that tested the efficacy of daily co-trimoxazole prophylaxis in reducing post-discharge mortality among HIV-uninfected children aged 60 days to 59 months admitted to hospital with SM in four hospitals in Kenya (two rural hospitals in Kilifi and Malindi, and two urban hospitals in Mombasa and Nairobi).^22^ Children were eligible for inclusion in the trial based on MUAC (<11.5 cm for children aged ≥6 months and <11.0 cm for infants aged 2–5 months) or presence of kwashiorkor; had a negative HIV rapid-antibody test; and had completed the stabilisation phase of treatment. Children were recruited into the trial for a median of 6 days from admission to the hospitals. Children were actively followed up for 1 year, monthly in the first 6 months and every 2 months until month 12 for death, readmission and growth, and traced at home if they defaulted study follow up visits. Samples were stored at -80°C until analysis.

### Variables and data source/measurement

The presence of oedema was evaluated by trained clinical research staff. Kwashiorkor was diagnosed based on the presence of bilateral pitting oedema regardless of concurrent wasting. Oedema severity in the discovery cohort was graded as: “+” – oedema on both feet; “++” – oedema in both feet and legs; “+++” – oedema in both feet, legs, arms, hands and face. Oedema severity was assessed by trained clinicians following World Health Organization guidelines.^23^ Oedema grade was not collected in the validation cohort. Children without nutritional oedema and with either mid-upper arm circumference <11.5cm (or <11cm if age <6 months) or weight-for-length/height (WFL/H) < -3 were considered as having marasmus.

### Study size

Of the 843 study children recruited in the F-75 trial, 209 (25%) children had oedema: grade one (+, n=67), grade two (++, n=113), and grade three (+++, n=29) at admission. A total of 181 (21%) oedematous children did not have albumin concentration measured at admission. Sample size was limited by finding exact matches of serum albumin concentrations between SM phenotypes to the nearest 1mg/L, and all matched case-control pairs were included. From the F-75 trial, 72 (19%) of children with nutritional oedema could be precisely matched on serum albumin concentrations to 72 (16%) children with marasmus.

The sample size calculation for the validation cohort was based on the results of the discovery cohort. Based on results for lumican (proteomics), we calculated that a sample size of 40 per group would be sufficient to achieve >80% power to find significant differences between the groups at α=0.05. Subjects for the validation cohort were selected if they had achieved nutritional and clinical rehabilitation (defined as having a MUAC >12.5 cm, absence of oedema and/or disease needing hospitalization) at day 60 post-hospital discharge. Selection was also limited to children who had sufficient plasma samples at enrolment and at month 2 of follow-up. Kwashiorkor cases (n=40) were matched to marasmus cases (n=40) on age, sex, and site of recruitment and randomisation arm in the trial.

### Untargeted plasma proteomics analysis

Liquid chromatography tandem mass spectrometry plasma proteomics analysis was performed for the discovery cohort using the plasma samples collected at enrolment during admission as described in our previous study.^24^

### Targeted plasma protein analysis

Plasma levels of syndecan-1 (Syn1) and hyaluronic acid (HA) were performed using quantitative ELISA (Thermo Scientific, MD, USA) following manufacturer's instructions at admission for the discovery cohort, and at admission and 60 days post-discharge for the validation cohort. Magnetic bead-based multiplex assay performed in a Luminex® platform (R&D Systems, MN, USA) were used to quantify plasma concentration of lumican, matrix metalloproteinase (MMP)2, tissue inhibitors of MMP (TIMP)1, TIMP2. These proteins were assayed at admission for the discovery cohort, and at admission and 60 days post-discharge for the validation cohort. Further, cytokine and chemokines (n=29) concentration in plasma at admission and 60 days post-discharge for the validation cohort were determined by using a human cytokine magnetic bead assay (EMD Millipore) on the Magpix with Xponent software (version 4.2; Luminex Corp) and acquired Median Fluorescent Intensity data analysed using the Milliplex analyst software (version 3.5.5.0 standard). Levels of CXCL-13, MMP3, 8, 13, and TGF-β were also measured but their concentrations were too low to be detected in most of the samples.

### Statistics

Baseline patient characteristics are provided as either mean ± standard deviation (SD), median (25 and 75th percentile) or proportions, as applicable. Difference in serum albumin concentrations across different grades of oedema severity was assessed by pair-wise comparison of means using linear regression adjusted for age, sex, HIV and site of recruitment. The probability of oedema formation in the entire F-75 trial was determined using binary logistic regression with presence or absence of oedema as outcome and serum albumin concentration as exposure. Change in serum albumin concentration after 3 days of inpatient treatment was determined using linear mixed modelling using serum albumin as outcome, time points as fixed effect and subject ID as random effect using the lmerTest package^25^ in R.

For the discovery cohort, difference in exposures (untargeted proteome and targeted metabolite levels, Syn1, HA, lumican, MMP2, TIMP1 and TIMP2, and a panel of 29 cytokines and chemokines) between marasmus and kwashiorkor were analysed using conditional logistic regression adjusted for age, sex, HIV status and recruitment site, to account for the sparseness introduced by exactly matching by serum albumin. For the validation cohort, differences between marasmus and kwashiorkor were analysed using conditional logistic regression to account for the sparseness introduced by exactly matching by age, sex and recruitment site. Conditional logistic regression was performed using the clogit function within the survival package in R (https://CRAN.R-project.org/package=survival).

To assess the association between increasing oedema severity and plasma/serum levels of glycocalyx components, individual protein and metabolite levels, we used an ordinal regression with age, sex, HIV status, recruitment site and serum albumin as additional covariates. High MUAC signifies the absence of wasting but MUAC can also increase with oedema in kwashiorkor.^26^ Hence, no adjustment for MUAC was made in the models as this may have obscured interpretation of the results. Longitudinal analyses were performed using linear mixed models with the individual subjects set as random effect using the lmerTest package^25^ in R. Correction for multiple testing was performed using Benjamini-Hochberg false-discovery rate method.^27^ Data analyses were performed in R version 3.6.1.

### Ethical approval

The F-75 trial was approved by the Kenya Medical Research Institute Ethical Review Committee (SCC 2799), College of Medicine Research Ethics Boards of the University of Malawi (P.03/14/1540), Oxford Tropical Research Ethics Committee (OXTREC 58–14) and the Hospital for Sick Children Research Ethics Board, Toronto (1000046559), including the secondary analysis in this manuscript. The co-trimoxazole trials was approved by Kenya Medical Research Institute National Ethical Review Committee (SSC 1562 and 2782) and Oxford Tropical Research Ethics Committee (reference number 18‐09), including the secondary analysis in this manuscript. Written informed consent was obtained from mothers or legal guardians of all study participants.

### Role of funding source

The funders of the study had no role in study design, data collection, data analysis, data interpretation, or writing of the report.

## Results

### Low serum albumin is necessary but not sufficient to develop oedema in SM

**Figure 1:**
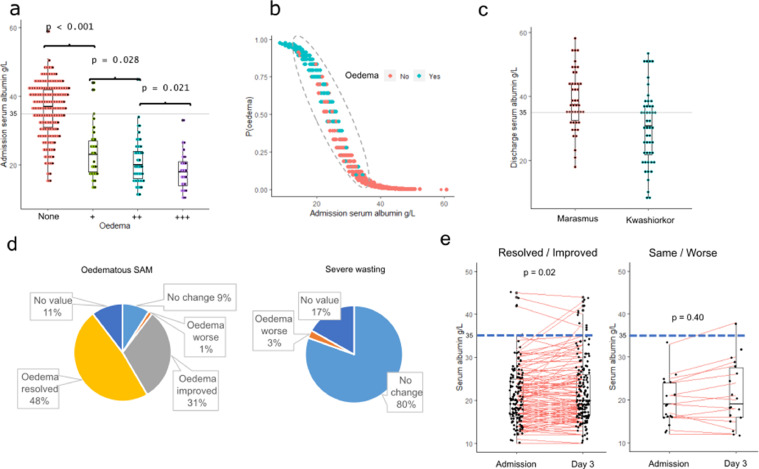
(a) Association between serum albumin concentration and degree of oedema severity in the F-75 reformation clinical trial: “None” means no oedema (marasmus) whereas “+” means oedema on both feet; “++” – oedema in both feet and legs; “+++” – oedema in both feet, legs, arms, hands and face. Oedema severity was assessed by trained clinicians following World Health Organization guidelines; (b) probability of presenting with oedema based on serum albumin concentration at hospital admission in the discovery cohort: green and red dots indicate those that presented with or without oedema respectively; (c) difference in serum albumin concentration between kwashiorkor and marasmus in the validation cohort; (d) distribution of oedema status after 3 days of hospitalization in the F-75 reformulation clinical trial; (e) changes in serum albumin concentration among kwashiorkor during admission and after 3 days of hospitalization.

We initially determined the association between serum albumin concentration and oedema using data from the entire F-75 trial among hospitalized children with severe acute malnutrition conducted in Malawi and Kenya.^21^ Within this trial, 79% (662/843) of the children had admission serum albumin data with a median (interquartile range; IQR) of 34g/L (IQR 24 – 40). HIV, older age, and enrolment at the Malawi site were associated with lower albumin concentrations, while breastfeeding, pre-existing heart disease, and presenting with severe pneumonia or diarrhoea were associated with higher albumin (all p<0.05) in multivariable analysis. Serum albumin concentration was negatively associated with the presence of oedema (aOR = 0.75 [95% CI: 0.71, 0.78] per g/L, p<0.001). We observed a significant decline in serum albumin concentration across increasing oedema severity grades according to the WHO classification (Figure 1a). Almost all children with oedema had serum albumin levels below 35g/L, but many children with similarly low levels of serum albumin did not have oedema or other features of kwashiorkor (Figure 1a,b). In the separate external validation cohort, children with SM recruited just prior to hospital discharge after stabilization in the co-trimoxazole trial in Kenya,^22^ serum albumin concentrations were significantly lower in children presenting with oedema compared to those without (Figure 1c) (aOR = 0.92 [95% CI: 0.87, 0.96], p = 0.001).

In the discovery cohort, oedema resolved within 3 days of hospitalization in 37 (48%) of children admitted with kwashiorkor, and oedema had an improved grade in 24 (31%) children. Two children (3%) among those admitted with marasmus developed oedema during treatment (Figure 1d). Among children whose oedema resolved or improved, there was a small increase in serum albumin concentration during the first 3 days of hospitalisation (0.68 g/L mean increase, p=0.02), whereas serum albumin remained unchanged among those whose oedema did not improve (Figure 1e). However, despite the small increase in serum albumin among children whose oedema resolved or improved, serum albumin concentrations at 3 days (median 20g/L, IQR 17–26g/L) remained far below clinically recognized norms in children (34–54g/L). Adjusting for regression to the mean indicated no differences in changes in serum albumin between all non-oedematous children recruited to this study and those admitted with oedema which either improved (p=0.93) or worsened (p=0.38). These findings strongly suggest that although low serum albumin is strongly associated with oedema in SM, additional factors are necessary to cause oedema.

### Selection of a sub-population matched on serum albumin levels

**Figure 2:**
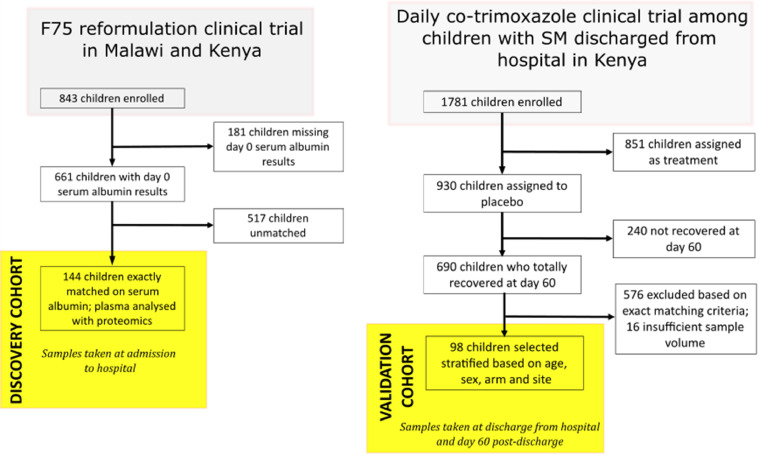
Recruitment flow diagram for the discovery and validation cohorts.

To determine factors associated with oedema in SM in conjunction with low albumin, we further selected children from the F-75 trial (Figure 2). This sub-cohort comprised children with kwashiorkor and marasmus matched on exact serum albumin levels and served as the discovery cohort for this study. In this matched discovery sub-cohort, age and sex distributions were similar in kwashiorkor and marasmus. MUAC was higher among children with oedema (p<0.001), whereas HIV was more prevalent among children without oedema (p<0.001). A greater proportion of kwashiorkor children were recruited in Malawi than in Kenya.

To validate the results obtained from the discovery sub-cohort, a validation sub-cohort was selected from the validation set from the co-trimoxazole trial in Kenya, with kwashiorkor and marasmus matched on age, sex, and site of recruitment, but not matched for serum albumin concentration (Figure 2). In the validation cohort, 51 and 47 children with and without oedema, respectively, were selected based on 25 strata. As with the F-75 cohort, serum albumin was higher in marasmus than in kwashiorkor children thus, for succeeding analyses, variables were normalized by serum albumin concentration in order to assess differences occurring independently of serum albumin concentrations to harmonize approaches with the discovery cohort. Normalization was done by presenting protein concentrations on a per g serum albumin basis. The baseline description of both cohorts is presented in Table 1 and flowcharts of the selection for both cohorts are presented in Figure 2.

**Table 1:** Admission characteristics of the children used in this study from the two independent cohorts.

	Discovery cohort			Validation cohort		
	Marasmus	Kwashiorkor	*p*ⅰ	Marasmus	Kwashiorkor	*p*ⅰ
*N*	72	72	/	47	51	/
Median age (mo.) at admission [IQR]	18 [12–27]	24 [15–31]	0.92	16 [13–24]	20 [17–25]	0.08
Girls *n* (%)	34 (47)	36 (50)	0.73	18 (38)	21 (41)	0.83
MUAC, cm mean ± SDⅱ	10.3 ± 1.4	11.8 ± 1.7	<0.001	10.8 ± 0.5	12.6 ± 0.9	<0.001
HIV status *n* (%) Positive Negative Unknown	36 (50) 32 (44) 4 (6)	14 (19) 54 (75) 4 (6)	<0.001	47 (100)	51 (100)	/
Weight-for-length z-score ± SD	-4.1 ± 1.5	/	/	/	/	/
Recruitment country *n* (%) Kenya Coast Provincial General Hospital Kilifi County Hospital Malindi sub-county Hospital Mbagathi Hospital Nairobi Malawi Queen Elizabeth Central Hospital	26 22 24	6 22 44	<0.001	26 4 5 12	29 5 5 12	1
Mean serum albumin (g/L) ± SDⅲ	24.4 ± 4.5	24.4 ± 4.5	1	38.6 ± 0.9	30.3 ± 1.2	<0.001

i Marasmus vs Kwashiorkor per cohort.

ii Mid-upper arm circumference.

iii Level at hospital admission for discovery cohort and level at hospital discharge for validation cohort.

**Figure 3:**
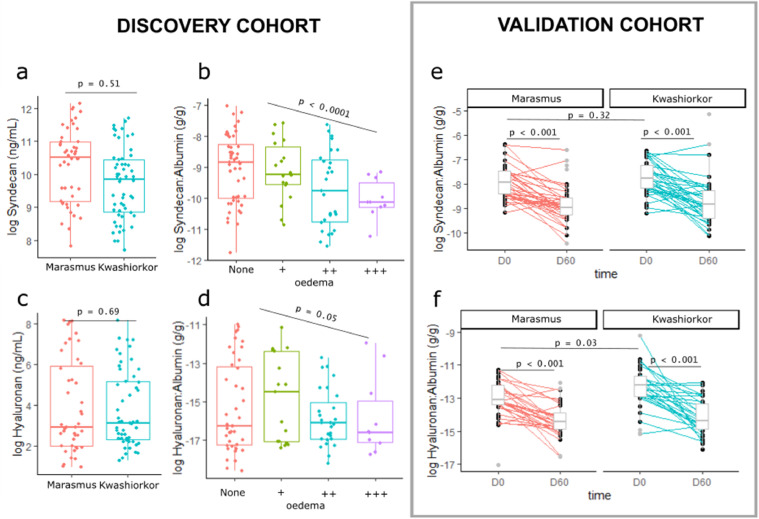
Association between endothelial plasma glycocalyx markers and kwashiorkor. (a,c) Association between the plasma concentrations of syndecan-1 and hyaluronic acid and kwashiorkor in the discovery cohort. (b,d) Association between the plasma concentrations of syndecan-1 and hyaluronic acid and the degree of oedema severity. “None” means no oedema (marasmus) whereas “+” means oedema on both feet; “++” – oedema in both feet and legs; “+++” – oedema in both feet, legs, arms, hands and face. Oedema severity was assessed by trained clinicians following World Health Organization guidelines; p values were estimated using ordinal logistic regression adjusted for age, sex, HIV status, and site of recruitment. Protein concentations were normalised by dividing with serum albumin concentration. (e,f) Association between the plasma concentrations of syndecan-1 and hyaluronic acid and kwashiorkor in the validation cohort in two time points: D0 is discharge from hospital, D60 is 60 days post-discharge.

Plasma markers of endothelial glycocalyx integrity are not associated with oedema in SM when comparing kwashiorkor and marasmus matched or normalised for serum albumin concentration. A reduced production of sulphated GAGs in kwashiorkor compared to marasmus has been previously suggested,^18^ indicating the potential role of EG integrity in kwashiorkor development. Therefore, we compared plasma markers of EG shedding among children with kwashiorkor and marasmus that were matched for serum albumin concentration. Given limited sample volumes, we prioritised the analysis of two abundant markers in plasma previously reported to be increased in diseases with known EG dysfunction^28^: Syn1, a proteoglycan bearing sulphated GAGs, and HA, a non-sulphated GAG. According to the revised Starling model and based on data in diseases associated with leakage of intravascular fluid and proteins leading to oedema, such as dengue,^28^ we initially hypothesized that kwashiorkor would be associated with increased plasma levels of Syn1 and HA. Surprisingly, neither Syn1 nor HA were associated with kwashiorkor compared to marasmus in the discovery cohort (Figure 3a,c). Their plasma levels were however negatively associated with increasing degree of oedema severity (Figure 3b,d). These results were replicated in the validation cohort, except for a modest but statistically (p = 0.03) higher plasma HA among children with kwashiorkor (Figure 3e, f). Plasma levels of Syn1 and HA significantly decreased after 60 days post discharge among children with either initial phenotype who fully recovered from malnutrition without further acute illness following discharge.

### Plasma extracellular matrix proteins are albumin-independent factors associated with oedema in SM

**Figure 4:**
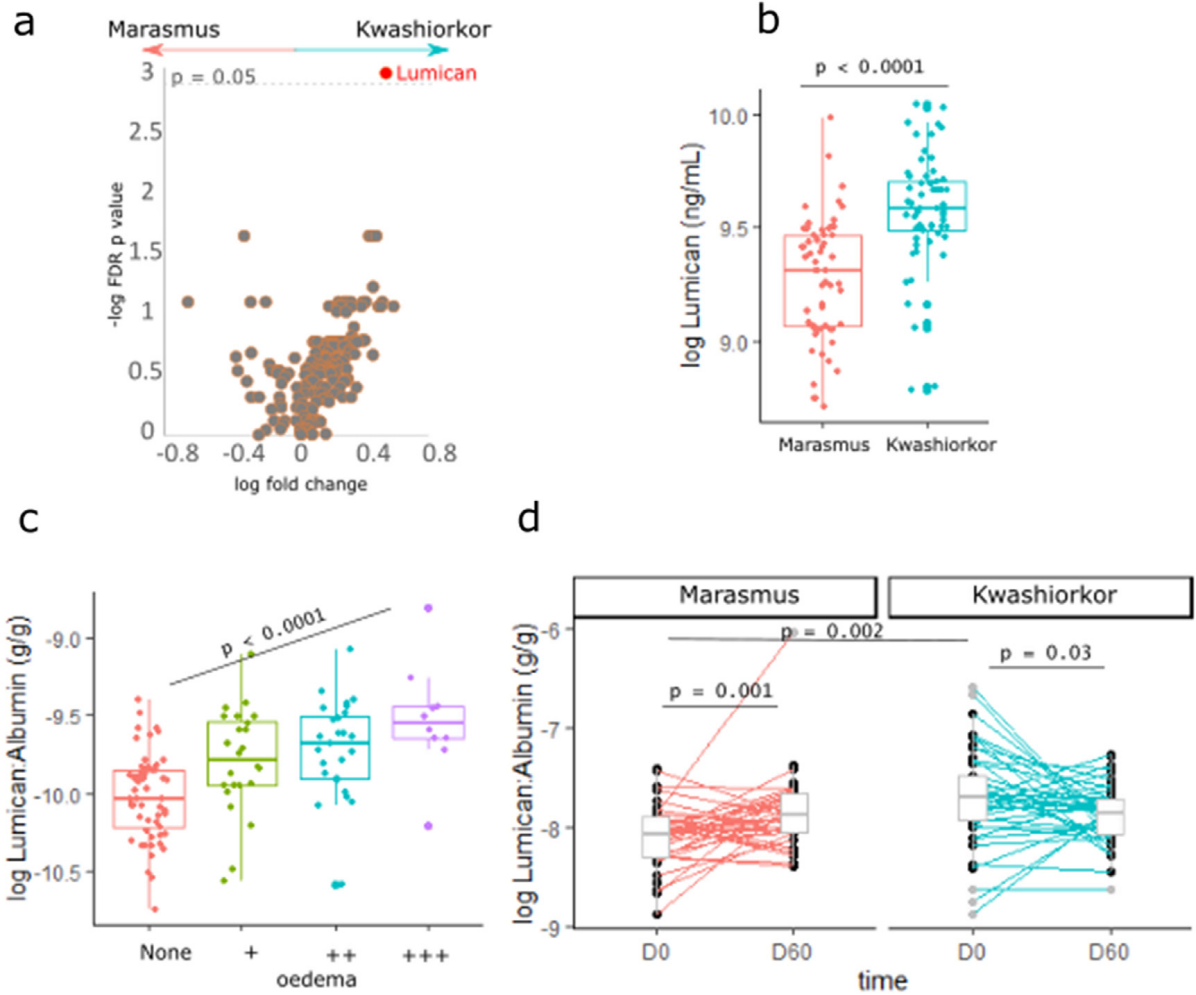
Differential abundance of proteins between kwashiorkor and marasmus. (a) Volcano plot showing the log fold change (x-axis) and –log p value after false-discovery rate adjustment of plasma proteins (y-axis). The horizontal line signify the FDR p = 0.05. Estimates were obtained using conditional logistic regression adjusting for age, sex, HIV status and site of recruitment stratified for admission serum albumin concentration. (B) Association between the plasma concentrations of lumican and kwashiorkor in the discovery cohort using ELISA. (c) Association between the plasma concentrations of lumican and the degree of oedema severity in the discovery cohort using ELISA. “None” means no oedema (marasmus) whereas “+” means oedema on both feet; “++” – oedema in both feet and legs; “+++” – oedema in both feet, legs, arms, hands and face. Oedema severity was assessed by trained clinicians following World Health Organization guidelines; p values were estimated using ordinal logistic regression adjusted for age, sex, HIV status, and site of recruitment. Protein concentations were normalised by dividing with serum albumin concentration.

To further discover albumin-independent mechanisms associated with oedema in SM, plasma samples from the discovery sub-cohort were subjected to untargeted proteomics analyses. Lumican was positively associated with kwashiorkor compared to marasmus cases matched by exact serum albumin levels (Figure 4a). We validated these findings using ELISA. Plasma lumican concentration by ELISA was found to be positively associated with kwashiorkor compared to marasmus (aOR = 1.49 [95% CI: 1.23, 1.79]) per µg/mL) (Figure 4b), and was positively associated with increasing degree of oedema severity (p<0.001) (Figure 4c).

The positive association of plasma lumican and kwashiorkor was replicated in the validation cohort for lumican levels normalized on serum albumin concentration (p=0.002) (Figure 4d). Comparing circulating lumican levels between discharge (still SM but free of underlying infections) and 60 days post-discharge (fully recovered from SM and infections) in the validation cohort, lumican was found to significantly increase (p<0.001) among children admitted with marasmus, whereas it decreased among children admitted with kwashiorkor (p<0.03), indicating a recovery of plasma lumican levels in both phenotypes after clinical and nutritional rehabilitation.

### Increased ECM degradation is an albumin-independent mechanism in oedema formation in SM

**Figure 5:**
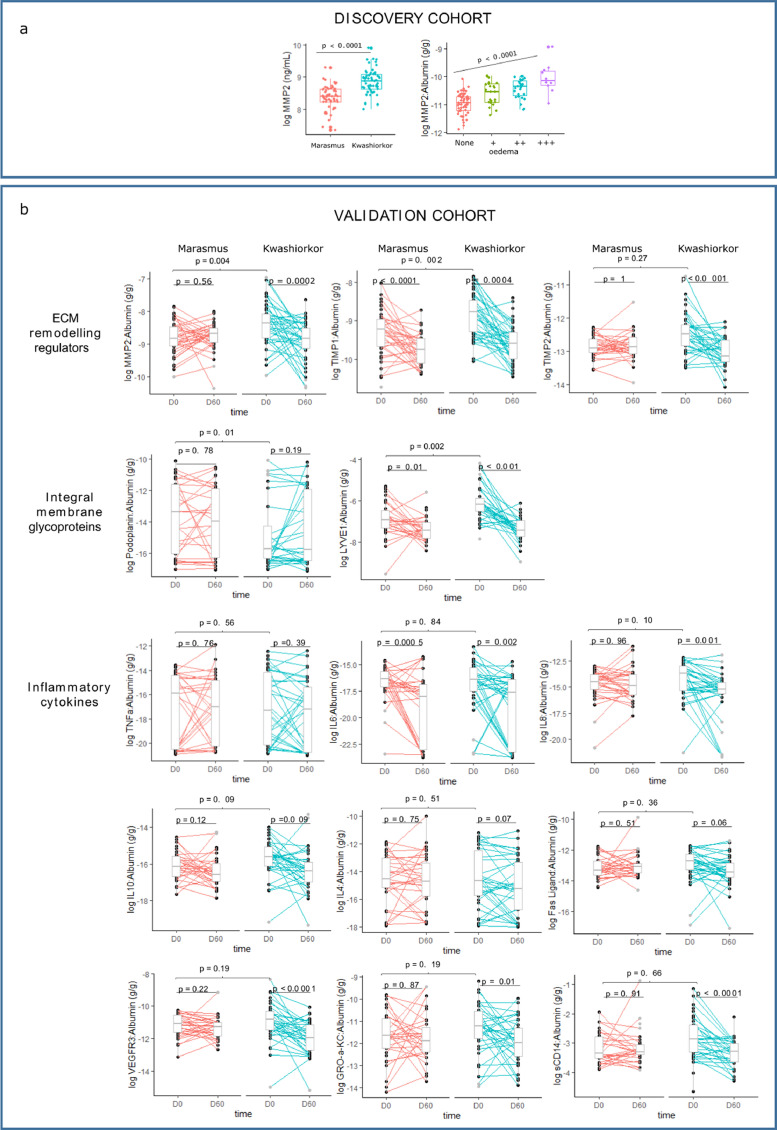
Association between SM phenotype and markers of ECM remodelling markers and systemic inflammatory. (a) association between MMP2 and SM phenotype, and severity of oedema in the discovery cohort. (b) associations between SM phenotype and ECM proteins and inflammatory cytokines in the validation cohort in two time points: D0 is discharge from hospital, D60 is 60 days post-discharge.

To further investigate the role of ECM degradation on oedema in SM, we compared plasma MMP2 concentrations between children with kwashiorkor and marasmus. Plasma MMP2 in the discovery cohort had a positive association with kwashiorkor (aOR = 1.89 [95% confidence interval: 1.38, 2.58] per µg/mL), and was associated with severity of oedema (p<0.001) (Figure 5a). Because of this, we measured plasma levels of ECM remodelling regulators, i.e. MMP2, tissue inhibitors of MMP (TIMP)1, and TIMP2, both at hospital discharge and at day 60 post-discharge among children who achieved full nutritional recovery. ECM remodelling regulators MMP2 and TIMP1 were positively associated with kwashiorkor. Furthermore, plasma levels of these proteins significantly reduced during nutritional rehabilitation (Figure 5b).

Based on the revised Starling model, filtered fluid in the interstitial space is drained back to the circulation mainly as lymph. Hence, we next measured ECM proteins found in the lymphatic system. Podoplanin, a mucin-like glycoprotein found in the alveoli, heart and lymphatic vascular system, was negatively associated, whereas lymphatic vessel endothelial HA receptor 1 (LYVE1) was positively associated with kwashiorkor. Plasma levels of LYVE1 also reduced during nutritional rehabilitation in both the kwashiorkor and marasmus phenotypes, but podoplanin remained unchanged.

### ECM degradation occurs in kwashiorkor despite similar plasma levels of inflammatory cytokines with marasmus

As ECM remodelling is activated by inflammation, we further measured key plasma markers of systemic inflammation in both SM phenotypes. None of the inflammatory cytokines measured were differentially abundant between kwashiorkor and marasmus cases matched for serum albumin, suggesting inflammatory response-independent mechanisms were driving ECM degradation in kwashiorkor (Figure 5b).

## Discussion

The conventional Starling model relied on the hypothesis that fluid filtration occurs at the arterial section of the capillaries under a dominant capillary hydrostatic pressure (*P_c_*) over interstitial hydrostatic pressure (*P_is_*), with fluid reabsorption occurring at the venous end due to *π*_c_ > *π*_is_.^6^ A balance of these forces is therefore necessary to keep fluid homeostasis and disruption of this balance, either increased filtration (*P_c_* - *P_is_*) or reduced reabsorption (*π*_c_ - *π*_is_), leads to fluid redistribution.

Albumin as the most abundant plasma protein, is the main driver of *π*_c_. Hence, in the absence of pathologies altering *P_c_*, a relationship between oedema and hypoalbuminaemia is often observed. However, the revised Starling model emphasizes on the contributions of other factors in fluid balance including the EG, the endothelial basement membrane, and the ECM. More importantly, Levick and Michal^7^ indicate that the main control of the interstitial volume is the activity of lymph flow which depends on the lymphatic function. Thus, hypoalbuminaemia is not the only determinant of oedema formation, as other factors within the intravascular, interstitium, and lymph compartments are likely involved. The role of the intravascular, interstitium, and lymph compartments in oedema formation in kwashiorkor (hypoalbuminemia) has not been well characterised. Here, we used children with oedematous and non-oedematous phenotypes of SM to understand mechanisms driving oedema formation. Our results demonstrated that oedema in SM is associated with both albumin-dependent and independent mechanisms.

Our data from two independent cohorts indeed show that serum albumin is lower among oedematous children and negatively associated with the degree of oedema severity. These data provide physiological support for the current WHO nutritional oedema grading system.^23^ There were almost no cases of kwashiorkor in whom serum albumin concentrations were more than 35 g/L in the discovery cohort. In the validation cohort who were enrolled towards the end of their hospital admission having initiated therapeutic feeding and no longer suffering acute infection, 35% of kwashiorkor cases had serum albumin levels above 35 g/L compared to 67% for the marasmus cases. We also observed that resolution or improvement of oedema was accompanied by a small increase in serum albumin concentrations, agreeing with previous observations.^1^ This initial small increase however is unlikely to be clinically relevant. Overall, these results indicate that low circulating albumin concentrations is necessary but is not sufficient to cause oedema in SM.

An alternative hypothesis for the aetiology of oedema in SM is a defect in sulphur metabolism leading to reduced production of sulphated GAGs that comprise the EG layer.^17^^,^^18^ In this study, we have shown that plasma levels of markers of EG shedding are not associated with oedema in SM, contrary to what would be expected, or to what is observed in other oedematous diseases, such as sepsis^29^ and nephrotic syndrome.^30^ We argue that EG shedding is associated with overall disease severity, including concurrent infection and inflammation, and not specifically oedema formation. Data of reduced GAGs in biological samples from children with kwashiorkor compared to marasmus could be attributed to the difference in clinical severity of the children upon sampling. It is also worth noting that in many of these studies,^31^, ^32^, ^33^, ^34^ children with kwashiorkor were compared to healthy controls and not with marasmus. The study of Amadi et al.,^18^ although presenting very important data, was performed on children with persistent diarrhoea who are eligible for intestinal biopsy. Hence, these children may not represent SM in general.

The ECM is a web of collagen fibrils within the interstitium composed of glycoproteins such as fibronectin and proteoglycans (proteins with GAG side chains).^8^ We found evidence of higher ECM degradation in children with kwashiorkor compared to marasmus evidenced by higher plasma levels of ECM proteins and ECM-remodelling enzymes. Lumican is a leucine-rich proteoglycan with keratan sulphate side chains and is a major component of corneal, dermal and muscle connective tissue. Downregulation of lumican results in skin fragility and laxity, and corneal opacity.^35^ Hence, degradation of lumican could explain the skin changes^36^^,^^37^ and corneal opacity^38^^,^^39^ reported to occur in some children with kwashiorkor. Although, no study has linked lumican specifically with the types of skin changes in kwashiorkor, i.e. “flaky paint” or “peeling paint” dermatosis, reduction of lumican levels have been reported in skin diseases such as actinic keratosis and Bowen's disease.^40^ On the other hand, LYVE1 and podoplanin are both markers of lymphatic endothelial integrity predominantly expressed in lymphatic vessels.^41^, ^42^, ^43^ LYVE1 and podoplanin are essential for lymphatic system development and ablation of podoplanin and LYVE1 in transgenic mice resulted in diminished lymphatic transport and lymphedema.^44^^,^^45^ Hence, differential plasma levels of these markers indicate that lymphatic system may be compromised in kwashiorkor, leading to poor lymphatic fluid drainage. Moreover, increased levels of plasma ECM were accompanied by increased plasma concentrations of MMP2, which is an active regulator of ECM remodelling. MMP2 is a 72 kDa type IV collagenase that is distributed in many tissues and associated with several serious diseases. Along with MMP9, it is also expressed in lymphatic endothelial cells^46^ and plays a key role in lymphatic vessel formation.^47^

The most important aspect of the revised Starling model is the mechanism of fluid reabsorption in which fluids are reabsorbed back to the circulation only via the lymphatic system.^7^ This implies that the lymphatic system plays a key role in protecting against oedema, as it is now regarded as the only route to remove excess fluid from the interstitial tissues. For instance, it has been previously reported that an increase in lymphatic flow maintains the plasma-to-interstitial protein ratio close to normal and thus protect against oedema in nephrotic syndrome patients with hypoalbuminaemia.^48^ Our study provides novel data implicating a damaged lymphatic drainage among kwashiorkor possibly due to degradation of ECM, leading to interstitial fluid expansion and oedema formation.

On the other hand, the ECM plays a more active role in maintaining *P_is_*. Conformational changes induced in the ECM allows the normally underhydrated GAG in the interstitium to expand and take up fluids. In inflammation, *P_is_* is reduced thereby increasing the transepithelial pressure difference leading to increased *J_v_* by as much as 20-fold.^49^ Golden have postulated that conformational changes and alterations in the interstitium may lead to its collapse from a gel generating the free liquid phase (free fluid of oedema) and the compact phase.^2^^,^^17^ However, although both mechanisms, i.e. ECM conformational change and impaired lymphatic drainage, could co-occur in kwashiorkor, it is more plausible that an impaired lymphatic drainage would contribute more towards interstitial fluid accumulation. This merits further investigation.

Therefore, independently of EG shedding, the ECM can play an active role in oedema formation in SM. The ECM is a highly dynamic structure in which components are continuously synthesized, degraded and regenerated,^50^ potentially explaining the resolution of oedema despite minimal increase in serum albumin observed among children recovering from kwashiorkor.

Despite known associations between inflammation and ECM degradation, we did not find differences in circulating inflammatory cytokines between children with kwashiorkor and marasmus. Thus, children with kwashiorkor may be predisposed to more ECM degradation compared to marasmus when exposed to the same level of inflammatory insult. Whether genetic, epigenetic or through dietary exposures, predisposition to an oedematous phenotype when exposed to SM and inflammation requires further investigation.

We reveal important findings on the pathophysiology of oedema formation relevant to SM and to other oedematous diseases. We recognize that matching patients by admission serum albumin for the specific objective of examining albumin-independent mechanisms precludes comparison with the full spectrum of marasmus cases, however that was not our intention. Hence, the discovery population could not be considered representative of the wider marasmus population as we focused on children with low serum albumin. Nonetheless, our results remained consistent in the validation cohort which was not limited to children with low serum albumin, it is more representative of SM in general and where we normalized by albumin rather than individually matched. Despite applying a rigorous study design by matching case-control pairs, we also recognise that sample size is relatively small in both discovery and validation cohorts.

Several other clinical complications are associated with altered the hydrostatic pressure gradient (*P_c_-P_is_*) leading to oedema, such as congenital heart disease, heart failure or nephrotic disease.^4^^,^^7^^,^^8^ Unfortunately, we lack detailed data on kidney and cardiac function in these children, which means we could not exclude effect of these potential pathologies in oedema development.

In conclusion, we have shown that oedema formation in SM has both albumin-dependent and independent mechanisms. ECM integrity appears to have a greater role in oedema formation than EG shedding in SM. Moreover, we provide novel data implicating a damaged lymphatic system in kwashiorkor. Further research is needed to understand the mechanisms driving increased ECM degradation, especially in the lymphatic system, in oedematous diseases.

### Data sharing statement

The processed data and R code may be accessed at the KEMRI-Wellcome data repository on the Harvard Dataverse under the Biosciences Dataverse subtheme (access here: https://dataverse.harvard.edu/dataset.xhtml?persistentId=doi:10.7910/DVN/NQCN2E). The mass spectrometry raw files generated and analysed in the current study have been deposited to the ProteomeXchange Consortium^51^ (PXD026477), via the MassIVE partner repository (MSV000087570), under the following title: “Albumin dependent and independent mechanisms in the syndrome of kwashiorkor”.

### Contributors

GBG, JMN and JAB conceived and planned the experiments. GBG, JMN, BMG and BW carried out the experiments. GBG, JMN and MS performed the data analysis. IP, JT, WV RHJB and JAB contributed to the biological sampling and supervision of the clinical trials. DL, JV, WV, RHJB and JAB contributed to the interpretation of results. GBG and JMN took the lead in writing the manuscript. All authors provided critical feedback and comments on the drafts of the manuscript. GBG and JMN contributed equally to this work (listed alphabetically).

### Funding

GBG was a postdoctoral fellow of the Research Foundation Flanders (FWO) and received financial support from the Thrasher Foundation Early Career Award (15122) and VLIR-UOS-Ghent University Global Minds Fund. Support for JMN, WV, JT, MN, and J.A.B. were provided by the Bill & Melinda Gates Foundation to JAB (Grant Number OPP1131320). JAB is also supported by the MRC/DfID/Wellcome Trust Global Health Trials Scheme (Grant Number MR/M007367/1). RHJB is supported by the Canadian Institutes of Health Research (156307). BW is supported by the Research Training Competition (RESTRACOMP) Graduate Scholarship at the Hospital for Sick Children and the Ontario Graduate Scholarship (OGS) at the University of Toronto. The F-75 reformulation trial was funded by Thrasher Research Fund (9403) while the co-trimoxazole trial was funded by the Wellcome Trust (WT083579MA), both awarded to JAB. This paper is published with the permission of the Director of the Kenya Medical Research Institute.

## Declaration of interests

None of the authors has a relevant conflict of interest.
